# Association of Medicaid Managed Care Drug Carve Outs With Hepatitis C Virus Prescription Use

**DOI:** 10.1001/jamahealthforum.2021.2285

**Published:** 2021-08-27

**Authors:** Samantha G. Auty, Paul R. Shafer, Stacie B. Dusetzina, Kevin N. Griffith

**Affiliations:** 1 Department of Health Law, Policy and Management, Boston University School of Public Health, Boston, Massachusetts; 2 Department of Health Policy, Vanderbilt University School of Medicine, Nashville, Tennessee

## Abstract

**Importance:**

Medicaid enrolls a disproportionate share of US adults with hepatitis C virus (HCV), and most receive Medicaid benefits through managed care organizations (MCOs). Medicaid MCOs often impose stricter requirements to access HCV medications than traditional fee-for-service Medicaid, which may inhibit use. Though Medicaid MCOs generally cover prescription drugs, several states have carved out direct-acting antiviral HCV medications from MCO coverage and opted to cover them under fee-for-service. Whether these carve outs were associated with changes in medication use is unknown.

**Objective:**

To examine the association between Medicaid-covered HCV medication fills and carve outs of these medications from MCO coverage.

**Design, Setting, and Participants:**

This cross-sectional study examined changes in fills of Medicaid-covered direct-acting antiviral HCV medications in 4 states (Indiana, Michigan, New Hampshire, and West Virginia) that carved out these drugs from Medicaid MCOs between 2015 and 2017. A synthetic control approach was used to compare changes in HCV prescription fills between states that did and did not carve out these medications from MCO prescription drug coverage. Data of direct-acting antiviral HCV prescription fills were obtained from the Medicaid State Drug Utilization Data files, January 2015 to June 2020. Data analysis was conducted from November 2020 to June 2021.

**Exposures:**

Carve outs of direct-acting antiviral HCV medications from Medicaid MCO prescription drug coverage.

**Main Outcomes and Measures:**

Direct-acting antiviral HCV prescriptions filled per 100 000 Medicaid enrollees.

**Results:**

In this cross-sectional study, carve outs were associated with a mean quarterly increase of 22.1 (95% CI, 12.7-34.1) HCV prescriptions per 100 000 Medicaid enrollees, a relative increase of 86.3% compared with synthetic control states. Compared with each state’s respective synthetic control, HCV prescription fills were associated with an increase of 11.5 (95% CI, 5.1-19.0) HCV prescription fills per 100 000 Medicaid enrollees per quarter in Indiana, 36.6 (95% CI, 23.5-53.9) in Michigan, 20.7 (95% CI, 11.1-32.8) in West Virginia, and 43.6 (95% CI, 25.9-68.4) in New Hampshire.

**Conclusions and Relevance:**

In this cross-sectional study of data from 39 states and the District of Columbia, carve outs of direct-acting antiviral HCV medications from Medicaid MCO prescription drug coverage were associated with significant increases in HCV medication use. Given their clinical benefits, greater uptake of HCV medication may help improve the health of Medicaid enrollees with HCV and reduce the economic burden of untreated HCV on the US health care system.

## Key Points

**Question:**

Did the use of direct-acting antiviral hepatitis C (HCV) medications change after these medications were carved out from Medicaid managed care organization (MCO) coverage and financed fee-for-service in 4 state Medicaid programs?

**Findings:**

In this cross-sectional study, carve outs were associated with a mean quarterly increase of 22.1 HCV prescription fills per 100 000 Medicaid enrollees compared with synthetic control states, translating to a relative increase of 86%.

**Meaning:**

This study suggests that carve outs of HCV medications from Medicaid MCO coverage may increase access to these medications, potentially improving the health of Medicaid enrollees with HCV and reducing the economic burden of untreated HCV on the US health care system.

## Teaser

This cross-sectional study assesses whether hepatitis C medication use would change in states that transitioned from carved-in to carved-out Medicaid coverage.

## Introduction

An estimated 4.1 million people are infected with the hepatitis C virus (HCV) in the US, and the prevalence of HCV is disproportionately higher among those enrolled in Medicaid.^1,2^ Since late 2013, the use of highly active direct-acting antivirals has revolutionized the treatment of HCV.^3^ In contrast with earlier HCV treatments, these newer medications are well tolerated and often curative after 1 course of treatment.^4^ The Infectious Diseases Society of America and American Association for the Study of Liver Diseases recommend treatment for all patients with acute or chronic HCV infection regardless of stage of infection.^5^ Timely treatment of HCV may avoid negative health consequences and reduce the economic burden of untreated HCV in the US health care system.^2^ Despite the effectiveness of HCV medications, state Medicaid programs often limit access to HCV medications because of their high cost, with list prices ranging from $25 000 to $95 000 for a single course of treatment.^6,7^ Historically, access to HCV treatment was limited through prior authorization requirements and restrictions requiring advanced liver damage and abstinence from use of substances.^8^ Despite access restrictions, HCV medications accounted for 5.1% of gross Medicaid spending on outpatient drugs ($3.2 billion) but only 0.02% of prescriptions in 2017.^9^

Medicaid managed care organizations (MCOs) deliver insurance coverage to more than 70% of Medicaid enrollees in the US through private health plans.^10^ Although federal regulation requires that Medicaid MCO prescription drug coverage be consistent with fee-for-service (FFS) plans (42 CFR § 438.210),^11^ MCOs often impose more stringent barriers to access HCV medications, including stricter clinical requirements or narrower preferred drug lists.^12,13^ These restrictions may reflect the financial risk faced by MCOs, which receive capitated payments from states for a defined package of benefits and the delivery of services to MCO enrollees.^14^ Comprehensive MCOs generally cover both inpatient and outpatient services and include prescription drug benefits in most states.^14,15^ However, a growing number of states have begun to selectively carve out direct-acting antiviral HCV medications from MCO coverage and finance them under FFS state Medicaid programs.^10,15,16^ Given differences in the utilization management techniques used by MCOs vs FFS Medicaid programs, carve outs of HCV medications from Medicaid MCO drug coverage may reduce access barriers and subsequently increase use of these lifesaving medications.^17^

To our knowledge, this is the first study to examine whether MCO carve outs of direct-acting antiviral HCV medications are associated with changes in use of HCV medications. We examined HCV medication use in 4 states (Indiana, Michigan, New Hampshire, and West Virginia) before and after these drugs were carved out from MCO coverage. We hypothesized that HCV medication use would increase in among states that transition from carved-in to carved-out coverage compared with a set of synthetic control states that did not experience changes in their mechanism of coverage of HCV medications.

## Methods

### Data and Sample

Data on Medicaid-covered HCV prescriptions were obtained from the Centers for Medicare & Medicaid Services Medicaid State Drug Utilization Data files from January 2015 to June 2020.^18^ States must report quarterly Medicaid-covered prescription volume to Centers for Medicare & Medicaid Services as a condition of their participation in the Medicaid Drug Rebate Program. Direct-acting antiviral HCV medications were identified using National Drug Codes according to methods used in previous research. eTable 1 in the Supplement presents a list of National Drug Codes.^4,19^ The Medicaid State Drug Utilization data include prescriptions that are eligible for the Medicaid Drug Rebate Program and thus do not include prescriptions financed under the 340B Drug Pricing Program.^18^

The HCV medication carve outs were identified using reports from the Kaiser Family Foundation.^16^ We confirmed effective dates of carve outs via state filings. We identified restrictions imposed by state Medicaid programs (FFS and MCO) on HCV medication access from a collaboration between the Center for Health Law and Policy Innovation at Harvard Law School and the National Viral Hepatitis Roundtable.^20^ We obtained data on HCV-related deaths from the Centers for Disease Control and Prevention WONDER database.^21^ We identified HCV-related deaths using *International Statistical Classification of Diseases and Related Health Problems, Tenth Revision (ICD-10)* codes 17.1 and 18.2 listed as either the underlying or additional cause of death. We obtained data on the incidence of acute HCV infections from the Centers for Disease Control and Prevention National Notifiable Diseases Surveillance System.^22^

We included states in our analysis if they had at least 1 year of data before an HCV medication carve out, provided criteria for HCV medication access publicly, and did not implement subscription-based payment models during the study period.^23^ South Carolina carved out HCV medication coverage in 2015 but was excluded because we required at least 1 full year of preperiod data. Illinois was excluded because several of the state’s largest Medicaid MCOs do not provide criteria for HCV medication access publicly. Louisiana and Washington were also excluded because they implemented subscription-based payment models for HCV medications in July 2019. Records (state-quarter National Drug Code) with fewer than 11 prescriptions are suppressed because of risk of patient identification, thus we excluded 7 states in which more than 50% of observations were suppressed (Alaska, Delaware, Iowa, Nebraska, North Dakota, South Dakota, and Wyoming). Five states were missing data for a single quarter (New Hampshire, West Virginia, South Carolina, Indiana, and Michigan), which we replaced through linear interpolation. Our final sample included 39 states and the District of Columbia, and our study period ranged from the first quarter of 2015 through the second quarter of 2020. Our unit of analysis was the state-quarter (880 state-quarters).

The Boston University Medical Center institutional review board determined that the need for approval was waived because the study was not human participants research. This study followed the Strengthening the Reporting of Observational Studies in Epidemiology (STROBE) reporting guideline for cross-sectional studies. Quarterly Medicaid enrollment was obtained from the CMS Monthly Enrollment Reports.^24^

### Primary Exposure

Our primary exposure was a binary indicator for whether HCV medications were carved out from state Medicaid MCO coverage. Indiana, Michigan, and New Hampshire carved out HCV medications from MCO prescription drug coverage in 2016, and West Virginia carved out coverage of all prescription drugs from MCO benefits in 2017. Effective dates are presented in eTable 2 in the Supplement.

### Primary Outcome

Our primary outcome was use of Medicaid-covered direct-acting antiviral HCV prescriptions, expressed as the quarterly rate of HCV medication fills per 100 000 Medicaid enrollees.

### Covariates

State Medicaid programs frequently impose criteria that require that enrollees have fibrosis (ie, liver damage) and/or sobriety to access HCV medications. We included these restrictions as covariates given their association with use of HCV medications.^25,26^ We considered states requiring any level of liver damage to access HCV medications as having a fibrosis requirement. Similarly, we considered states requiring any period of substance abstinence as having a sobriety requirement. Fibrosis and sobriety requirements were coded as binary variables taking on a value of 1 if a state had any restrictions in place during a quarter, and 0 otherwise.^20^

### Statistical Analysis

We used synthetic control methods to estimate the association between carve outs and use of HCV medications, which compare outcomes in a treated state to a weighted average of control states by matching on preperiod trends and other covariates.^27,28^ Synthetic control methods are particularly useful when estimating how a policy change may affect a small number of treatment groups at varying times,^27,28,29,30,31^ and build on traditional difference-in-differences estimation but require fewer assumptions.^27,32^ For example, difference-in-differences assumes that any differential changes in outcomes between treated and control groups are attributable to the policy change. Yet treated and control groups are often nonequivalent in terms of pretreatment outcome levels, trends in outcomes, and other important covariates. Nonequivalence of treatment and control groups introduces potential selection issues, which may bias estimates of association. Researchers using traditional regression approaches attempt to control for a wide range of potential confounders associated with either treatment assignment or study outcomes. In contrast, synthetic control methods mitigate this limitation by constructing a synthetic control from a donor pool of groups not exposed to the treatment of interest. Researchers may also include covariates in synthetic control methods, but they are not often necessary and may worsen the preperiod matches between treated states and their respective synthetic control.^27^ The synthetic control state is constructed using a weighted average of untreated states so that the synthetic control is similar to the treated state in outcome trends and other specified covariates pretreatment. In other words, instead of comparing New Hampshire (ie, treated group) to New York, Texas, California, or a simple average of those states (ie, untreated group), researchers can use synthetic control methods to construct a weighted average of these states so the resulting synthetic control is a better match to the treated state with respect to pretreatment characteristics and outcomes trends.^27^

Our analysis proceeded in 4 steps. First, we created individual synthetic control states for each treated state (ie, synthetic Indiana, synthetic Michigan, synthetic New Hampshire, and synthetic West Virginia) from weighted averages of states in the donor pool (untreated states).^32^ Weights were selected using a data-driven approach to minimize preperiod differences between treated states and their respective synthetic control states across 3 variables. These variables included liver damage and sobriety criteria for Medicaid-covered HCV treatment access, as well as HCV medication fills per 100 000 Medicaid enrollees. eTable 3 in the Supplement presents the actual weights used.

Second, we estimated mean differences in quarterly HCV prescription fills per 100 000 Medicaid enrollees between treated states and synthetic control states during the 2 years after treated states’ adoption of carve-out policies. This 2-year period was selected to provide adequate time for clinicians to respond to changes in coverage policies and to limit the risk that future health policy changes may confound our results.^33^ In sensitivity analyses, we tested a 1-year postperiod. We also visually assessed whether there were differences in trends in HCV-related death rates (expressed as the number of deaths per 100 000 residents) or infection incidence (expressed as the number of acute infections per 100 000 residents) among treated states during the preperiod.

Third, we used Taylor series linearization to determine 95% CIs and performed permutation tests because synthetic control methods do not produce traditional measures of uncertainty.^27,28^ Permutation tests were conducted by reassigning the treatment to each synthetic control state one by one, rerunning our synthetic control models, and estimating placebo effect sizes. If the observed effect sizes in treated states exceed the placebo effect sizes in synthetic control states during permutation tests, this further indicates that our results were unlikely to have occurred by chance. The number of permutation tests varied by state; individual permutation tests that could not achieve a suitable match during the preperiod were removed when calculating results. Specifically, states were removed from permutation tests if the mean squared error between the treated state and its synthetic control was greater than 1. Any mean squared error cutoff is by definition arbitrary; thus we also tested thresholds of 0.5 and 1.5 and achieved similar results. Note that unlike traditional regression analyses, the CIs are not typically symmetric around the point estimate. We then used a 2-sided *t* test to determine whether the observed effects in treated states exceeded the placebo effects in control states at the α = .05 significance level. If so, this provided further support that results were unlikely to have occurred by chance.

In addition, we created overall estimates of the association of carve outs with HCV prescription fills, achieved by weighting the individual state-level results by their mean Medicaid enrollment totals during the 2-year postperiod. Analyses were conducted using R version 4.0.2 (R Project for Statistical Computing), with synthetic control models estimated using the microsynth package version 2.0.17.^34^ Data analysis was conducted between November 2020 and June 2021. Additional details are available in the eMethods in the Supplement.

### Robustness Tests

One state (New Hampshire) carved out coverage of direct-acting antiviral HCV medications from MCO prescription drug benefits in 2016, then carved these medications back in during 2019. This situation allowed us to estimate changes in HCV medication use within a single state that moved from treated (carved out) back to untreated (carved in) during the study period. If our effect estimate is in the opposite direction of those found in analyses of carve outs, it lends further credibility to our results.

## Results

All states that carved out coverage of HCV medications enrolled the majority of Medicaid enrollees in managed care plans during the study period. Prior to carving out coverage of HCV medications 61% of enrollees in Illinois, 66% of enrollees in New Hampshire, 50% of enrollees in Michigan, and 71% of enrollees in West Virginia were enrolled in Medicaid managed care plans.^24^ Trends in HCV medication use were generally stable in treated states and were highly similar to those in synthetic control states prior to carve outs (Figure). Liver and sobriety restrictions remained consistent in all treated states during the 2-year period after adoption of carve-out policies except New Hampshire, which removed both restrictions when it carved out HCV medications from MCO coverage. Incidence of acute HCV infections and HCV mortality rates was relatively stable among treated states during the preperiod (eFigure 1 in the Supplement). The estimated differences in use between each treated state and its synthetic control state are presented in the Table, and HCV prescription fill trends in treated states and their respective synthetic control states are presented in the Figure.

**Figure.**
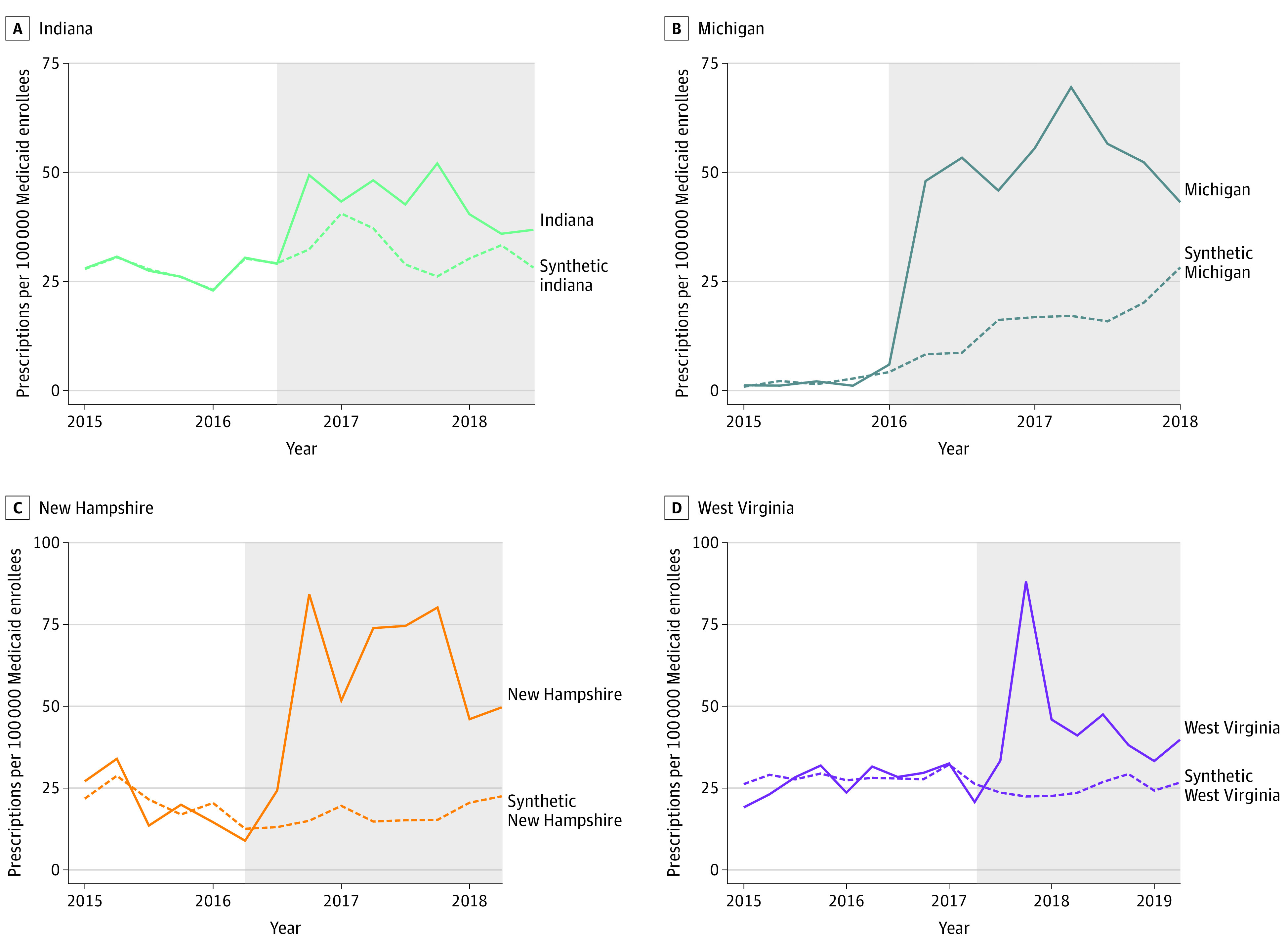
Trends of Medicaid-Covered Hepatitis C Prescription Fills per 100 000 Medicaid Enrollees in Treated vs Synthetic Control States The shaded area indicates the 2-year period after adoption of carve-out policies in each state. Weights used to construct the synthetic control states are presented in eTable 1 in the Supplement.

**Table.** Absolute Differences in HCV Prescription Fills Between Treated States and Synthetic Control States in the Period After Adoption of Carve-Out Policies

State	Policy change^a^		Treated^b^	Synthetic control^b^	Difference (95% CI)^c^
	Year	Quarter			
Overall	NA	NA	47.7	25.6	22.1 (12.7-34.1)
Indiana	2016	Q4	43.6	32.1	11.5 (5.1-19.0)
Michigan	2016	Q2	53.0	16.5	36.6 (23.5-53.9)
New Hampshire	2016	Q3	60.6	17.0	43.6 (25.9-68.4)
West Virginia	2017	Q3	45.7	25.0	20.7 (11.1-32.8)

Abbreviations: HCV, hepatitis C virus; NA, not applicable.

^a^ Year and quarter of carve out implementation.

^b^ Quarterly rates of HCV prescriptions per 100 000 Medicaid enrollees during the postperiod. Synthetic control states were also matched based on liver and sobriety restrictions. Estimates are for 2 years after the policy change.

^c^ Upper and lower bounds of confidence intervals were found using Taylor series linearization.

### Overall Results

The HCV medication carve outs were associated with an additional 22.1 quarterly prescription fills per 100 000 Medicaid enrollees in the 2 years following carve outs (95% CI, 12.7-34.1), a relative increase of 86.3% (95% CI, 49.6%-133.2%). State-by-state estimates are provided below.

### Indiana

In the year before the carve out, the mean (SD) rate of HCV prescription fills in Indiana was 26.5 (3.8) per 100 000 Medicaid enrollees per quarter. The mean (SD) rate of HCV prescription fills increased to 43.6 (5.9) per 100 000 in the post–carve-out period. We estimate that the carve out was associated with an additional 11.5 (95% CI, 5.1-19.0) HCV prescription fills per 100 000 Medicaid enrollees compared with synthetic Indiana, a relative increase of 35.7% (95% CI, 15.9%-59.0%). However, findings of similar magnitude were observed in a small number of permutation tests (3 of 25 tests; *P* = .15).

### Michigan

In the year before the carve out, the mean (SD) rate of HCV prescription fills in Michigan was 1.6 (0.6) per 100 000 Medicaid enrollees per quarter. This increased to a mean (SD) of 53.0 (8.1) HCV prescription fills per 100 000 in the post–carve-out period. We estimate that the carve out was associated with an additional 36.6 (95% CI, 23.5-53.9) HCV prescriptions per 100 000 Medicaid enrollees, a relative increase of 221.6% (95% CI, 142.2%-327.0%) compared with synthetic Michigan. Findings of similar magnitude were not observed in permutation tests (0 of 28 tests; *P* = .03).

### New Hampshire

In New Hampshire, the mean (SD) rate of HCV prescription fills per quarter was 16.0 (3.4) per 100 000 Medicaid enrollees before the carve out, which increased to 60.6 (20.9) in the post–carve-out period. The synthetic New Hampshire results suggest that carve outs were associated with an additional 43.6 (95% CI, 25.9-68.4) HCV prescriptions per 100 000 Medicaid enrollees, a relative increase of 256.2% (95% CI, 152.5%-402.4%). Findings of similar magnitude were not observed in permutation tests (0 of 34 tests; *P* = .03).

### West Virginia

The mean (SD) rate in West Virginia of HCV prescription fills during the preperiod was 30.3 (2.1) per 100 000 Medicaid enrollees, which changed to 45.7 (18.0) HCV prescription fills after the carve-out period. The carve out was associated with an additional 20.7 (95% CI, 11.1-32.8) quarterly HCV prescriptions per 100 000 Medicaid enrollees, a relative increase of 82.8% (95% CI, 44.5%-131.3%) compared with synthetic West Virginia. Findings of similar magnitude were observed in a small number of permutation tests (3 of 32 tests; *P* = .12).

### Robustness Checks

Synthetic control results show that carving in HCV medications in New Hampshire was associated with a decrease in the rate of HCV prescription fills compared with its synthetic control state (−22.0; 95% CI, –31.4 to –6.5), a relative decrease of 30.7% (95% CI, –46.8% to –9.7%). Findings of similar magnitude were not observed in permutation tests (0 of 22 tests; *P* = .045).

## Discussion

Carve outs of direct-acting antiviral HCV medications from Medicaid MCO drug coverage were associated with increased use of these drugs, which may lead to improved health and quality of life for Medicaid enrollees with HCV. Though states have largely transitioned delivery of Medicaid to MCOs to reduce their financial risk and gain budget predictability, it is important to ensure that the transition from FFS to MCO coverage does not decrease access to highly effective but expensive treatments. Given that insurance churn occurs frequently among Medicaid enrollees and MCOs assume full financial risk for the delivery of covered services, they are incentivized to limit use of high-cost services even if these treatments are high value in the long term.^35^ We found evidence for this in the 4 states that changed from carved-in to carved-out status for HCV medications during our study period as HCV prescription fills increased from 35.7% (Indiana) to 256.2% (New Hampshire) compared with their respective synthetic control states. Although permutation tests indicate that our results for Michigan and New Hampshire were unlikely to have occurred by chance, findings of similar magnitude were observed in some permutation tests for Indiana and West Virginia (eFigure 2 in the Supplement). Differences in HCV medication use between treated states and synthetic control states also appeared to narrow over time, which may reflect pent-up demand for HCV treatment that was unmet under carved-in benefits. Additionally, differences in the contractual arrangements between MCOs and states may moderate the association between carve outs and use. For instance, MCO contracts may limit profits and medical loss ratios to specific parameters, which may determine the degree of financial risk faced by MCOs when covering high-cost medications.

Carve outs of high-cost medications may thus represent an important strategy for states to increase access to these medications, especially when combined with other approaches. Under a capitated payment model, Medicaid MCOs face financial risk in delivering coverage of health care for their enrollees and have a limited set of strategies to mitigate this risk.^10^ Alternative coverage policies may enable Medicaid MCOs to increase access to high-cost medications. Kick payments, supplemental payments made by the state to cover specific services,^16^ may enable MCOs to reduce access restrictions by shifting financial risk of covering HCV treatment back to the state. Although kick payments are most commonly used for maternity-related services, they have also been implemented for high-cost medications.^16,36^

Another strategy to reduce restrictions imposed by Medicaid MCOs is for states to regulate and enforce coverage parity between MCO and FFS plans.^20^ Although several states require drug coverage parity between FFS and MCO plans, many states allow MCOs to impose stricter clinical criteria or prior authorization requirements to access HCV medications.^20^ Since 2014, 6 states have adopted uniform preferred drug formularies, which mandate that the same medications be covered without the need to obtain prior authorization for those enrolled in Medicaid managed care and FFS.^16^ Uniform preferred drug formularies may improve access to prescription medications by standardizing the drugs that MCO plans must cover to a statewide benchmark. Other strategies to enforce drug coverage parity include policies that require MCOs to impose no more restrictive criteria to access prescription medications than those imposed by Medicaid FFS.^16^ Many states that use MCOs to deliver coverage have implemented or plan to implement policies that encourage drug coverage parity, but no research has assessed how these policies affect access to prescription medications.

Innovative payment models for high-cost medications may offer a population health–centered solution that constrains per prescription costs while increasing access. Subscription-based payment models, wherein states negotiate reduced prices with a single drug manufacturer in exchange for exclusive inclusion on drug formularies, may reduce per prescription spending while increasing access.^37^ Louisiana and Washington adopted subscription-based payment models for HCV medications in 2019. HCV prescription fills increased substantially in Louisiana but did not change in Washington after implementation of subscription-based payment models.^38^ To date, no research has assessed the effect of subscription-based payment models on spending on HCV medications.

Timely treatment of HCV is paramount to reduce the burden of HCV-related morbidity and mortality.^3^ Although the short-term costs of treatment are high, reductions in negative health consequences associated with HCV may result in considerable cost savings in the long term.^2^ Moreover, by restricting access to curative HCV treatment, MCOs may be worsening long-term health outcomes among those with HCV.

### Limitations

This study has limitations. First, suppression of Medicaid-covered HCV prescriptions in predominantly rural states may limit the generalizability of our results. However, we were able to achieve an acceptable match in preperiod outcomes between our treated states and their respective synthetic control states. Second, the Medicaid State Drug Utilization Data do not include prescriptions financed under the 340B program, and our results may not generalize to prescriptions financed under this mechanism. Third, our data do not allow us to elucidate potential mechanisms underlying differential changes following carve outs in treated states. For instance, differences in HCV prevalence could affect state-level demand for HCV medications. These data were not available; however, we did not find evidence of differential preperiod trends between treated and synthetic control states for which data were available in either HCV infection incidence or mortality. Fourth, we are unable to disentangle removal of access restrictions and HCV medication carve outs, which occurred concomitantly in New Hampshire. In addition, the observational nature of our study design limits the causal conclusions regarding the effects of MCO carve outs on use of direct-acting antiviral HCV medications.

## Conclusions

In this cross-sectional study, carve outs of direct-acting antiviral HCV medications from Medicaid MCO coverage were associated with increased use of these medications. Medicaid MCOs may limit use of lifesaving HCV medications, potentially adversely affecting the health of Medicaid enrollees with HCV. States and MCOs should explore alternative strategies to limit spending on high-cost medications while ensuring widespread access. As states implement policies that attempt to constrain Medicaid spending on prescription medications, policy makers should consider potential unintended consequences for access.

## Notes

Supplement. **eTable 1.** National Drug Codes Used to Identify Direct-Acting Antiviral HCV Medications **eTable 2.** Policy Effective Dates for HCV Drug Coverage Changes in Medicaid **eTable 3.** States With Nonzero Weights in Synthetic Control Models
eMethods

eReferences
**eFigure 1.** Trends of Hepatitis C Mortality per 100 000 Residents in Treated States and Nationally **eFigure 2.** Trends in HCV Prescription Fills per 100 000 Enrollees per Quarter for Treated and Individual Control States
