# Supplementary material for: Association of Medicaid Managed Care Drug Carve Outs With Hepatitis C Virus Prescription Use
Source: JAMA Health Forum. Author manuscript; Available in PMC 2022 Feb 7. (PMC8796891; doi:10.1001/jamahealthforum.2021.2285)
Supplement: Online Supplement — eTable 1. National Drug Codes Used to Identify Direct-Acting Antiviral HCV Medications eTable 2. Policy Effective Dates for HCV Drug Coverage Changes in Medicaid eTable 3. States With Nonzero Weights in Synthetic Control Models eFigure 1. Trends of Hepatitis C Mortality per 100 000 Residents in Treated States and Nationally eFigure 2. Trends in HCV Prescription Fills per 100 000 Enrollees per Quarter for Treated and Individual Control States [file NIHMS1761373-supplement-Online_Supplement.pdf]

Supplemental Online Content

Auty SG, Shafer PR, Dusetzina SB, Griffith KN. Association of Medicaid managed care drug carve outs with antiviral hepatitis C prescription use. *JAMA Health Forum.* 2021;2(8):e212285. doi:10.1001/jamahealthforum.2021.2285

eTable 1. National Drug Codes Used to Identify Direct-Acting Antiviral HCV Medications

eTable 2. Policy Effective Dates for HCV Drug Coverage Changes in Medicaid

eTable 3. States With Nonzero Weights in Synthetic Control Models

eMethods

eReferences

eFigure 1. Trends of Hepatitis C Mortality per 100,000 Residents in Treated States and Nationally

eFigure 2. Trends in HCV Prescription Fills per 100,000 Enrollees per Quarter for Treated and Individual Control States

This supplemental material has been provided by the authors to give readers additional information about their work.

© 2021 Auty SG et al. *JAMA Health Forum*.

eTable 1. National Drug Codes used to identify direct-acting antiviral HCV medications.

Drug Name	Active Ingredients	11-Digit NDC
Sovaldi	Sofosbuvir	61958150101
Sovaldi	Sofosbuvir	61958150201
Sovaldi	Sofosbuvir	61958150202
Sovaldi	Sofosbuvir	61958150301
Sovaldi	Sofosbuvir	61958150401
Sovaldi	Sofosbuvir	61958150501
Harvoni	Ledipasvir/sofosbuvir	61958180501
Harvoni	Ledipasvir/sofosbuvir	61958180401
Harvoni	Ledipasvir/sofosbuvir	61958180301
Harvoni	Ledipasvir/sofosbuvir	61958180202
Harvoni	Ledipasvir/sofosbuvir	61958180101
Viekira Pak	Ombitasvir/paritaprevir/ritonavir/dasabuvir	00074309328
Viekira Pak	Ombitasvir/paritaprevir/ritonavir/dasabuvir	00074309301
Technivie	Ombitasvir/paritaprevir/ritonavir	00074308228
Zepatier	Elbasvir/grazoprevir	00006307402
Zepatier	Elbasvir/grazoprevir	00006307401
Epclusa	Sofosbuvir/velpatasvir	61958220101
Epclusa	Sofosbuvir/velpatasvir	61958220201
Epclusa	Sofosbuvir/velpatasvir	61958220202
Epclusa	Sofosbuvir/velpatasvir	61958220203
Epclusa	Sofosbuvir/velpatasvir	61958220301
Viekira XR	Dasabuvir/ombitasvir/paritaprevir/ritonavir	00074006328
Viekira XR	Dasabuvir/ombitasvir/paritaprevir/ritonavir	00074006301
Vosevi	Sofosbuvir/velpatasvir/voxilaprevir	61958240101
Mavyret	Glecaprevir/pibrentasvir	00074262528
Mavyret	Glecaprevir/pibrentasvir	00074262501
Mavyret	Glecaprevir/pibrentasvir	00074262556
Mavyret	Glecaprevir/pibrentasvir	00074262580
Epclusa authorized generic	Velpatasvir/sofosbuvir	72626270101
Harvoni authorized generic	Ledipasvir/sofosbuvir	72626260101

© 2021 Auty SG et al. *JAMA Health Forum*.

eTable 2. Policy effective dates for HCV drug coverage changes in Medicaid.

State	Effective Date	Policy Change	Post Period	URL
Michigan	3/1/2016	Carve-out	Q2, 2016 – Q2, 2018	https://bit.ly/3tc4HsH
New Hampshire	8/1/2016	Carve-out	Q3, 2016 – Q3, 2018	https://bit.ly/3ciK4UT
Indiana	9/1/2016	Carve-out	Q4, 2016 – Q4, 2018	https://bit.ly/3rBeeci
West Virginia	7/1/2017	Carve-out	Q3, 2017 – Q3, 2019	https://bit.ly/30vKKAB
New Hampshire	9/1/2019	Carve-in	Q4, 2019 – Q2, 2020	https://bit.ly/3rzConC

© 2021 Auty SG et al. *JAMA Health Forum*.

eTable 3. States with nonzero weights in synthetic control models

	Weight			
**Control state**	**Synthetic Indiana**	**Synthetic Michigan**	**Synthetic New Hampshire**	**Synthetic West Virginia**
Alabama	6.6	--	--	38.6
Arizona	--	1.6	--	--
Arkansas	--	12.4	35.8	--
Colorado	31.8	50.6	--	--
Florida	--	--	--	11.6
Georgia	45.5	--	11.6	9.4
Hawaii	3.9	--	--	--
Kansas	--	5.0	29.8	20.7
Maryland	--	--	--	2.9
Mississippi	--	--	--	1.2
Nevada	6.3	--	--	7.7
New Mexico	3.1	--	--	--
North Carolina	--	--	--	4.7
Rhode Island	2.7	--	--	3.0
Texas	--	2.1	--	--
Utah	--	--	18.7	--
Vermont	--	--	4.0	--
Wisconsin	--	28.3	--	--

Notes: The table displays the percentage that each state contributed to the synthetic control. Numbers may not sum to 100 due to rounding.
-- *indicate a state received no weight in that synthetic control model*

© 2021 Auty SG et al. *JAMA Health Forum*.

eMethods

Sample and Measurement of Outcome Variables

The pre-period for our analysis ran from January 1, 2015 to each states' respective effective quarter for MCO medication carve-outs, and our post-period was the two years following the carve-out effective quarter (see Appendix II for effective dates and post-periods for each state). We included states in our analysis if they had at least one year of pre-period data following a HCV medication carve-out, provided criteria for HCV medication access publicly, and did not implement subscription-based payment models during the study period. Justifications for the exclusion of specific states are discussed in the main text. Over final "donor pool" included the following: Alabama, Arizona, Arkansas, California, Colorado, Connecticut, District of Columbia, Florida, Georgia, Hawaii, Idaho, Kansas, Kentucky, Maine, Maryland, Massachusetts, Minnesota, Mississippi, Missouri, Nevada, New Jersey, New Mexico, New York, North Carolina, Ohio, Oklahoma, Oregon, Pennsylvania, Rhode Island, Tennessee, Texas, Utah, Vermont, Virginia, and Wisconsin.

Unadjusted Comparisons

Unadjusted changes in study outcomes were identified by comparing the four calendar quarters preceding carve-outs with the following eight quarters. Significance levels and confidence intervals were calculated using two-sided t-tests.

Synthetic Control Comparisons

The basic idea of synthetic control methods is to use a weighted average of each state's donor pool, with the weights chosen so that pre-trends in outcomes were as similar as possible between the treated state(s) and synthetic control.[1] We followed the approach outlined in Robbins, Saunders, and Kilmer (2016) and implemented in package 'microsynth' for R Statistical Software version 4.0.2.[2] We estimated separate synthetic controls models for each treated state and study outcome.

Taylor series linearization (TSL) was then used to calculate estimates and 95% confidence intervals for the effects of SBPM implementation during the following year. This approach accounts for the complex weighting of control states used to create the synthetic control. TSL uses a linear function of the observed data to approximate the estimator, and the variance estimation formulae for a linear estimator can then be applied to the linear approximation. In general, this leads to a statistical consistent estimator of the variance.[3] The TSL method tells us whether any differences in outcome between the treated states and their synthetic controls are statistically significant.

We also conducted a series of permutation tests to determine placebo effect sizes. This procedure occurs in three steps. First, we subset the donor pool of control states for a given treated state and outcome. We then iteratively reassign the "treatment" to each control state. Weights are then calculated to match the placebo treatment to a new synthetic control, and a placebo effect, sampling distribution and associated p-value are generated. Weights were selected to minimize not only outcome pre-trends, but also differences in state liver damage and sobriety restrictions during the pre-period. The number of permutation tests varied by state; individual permutation tests that could not achieve a suitable match during the pre-period were dropped when calculating results. This was defined as a mean squared error (MSE) between treated state and synthetic control of greater than 1. Any MSE cutoff is by definition arbitrary, thus we also tested thresholds of 0.5 and 1.5 and achieved highly similar results. We then use a two-sided t-test to determine whether the observed effect in the treated state is likely to have occurred by chance, given the distribution of placebo effect estimates.[4]

© 2021 Auty SG et al. *JAMA Health Forum*.

References

1. Athey S, Imbens GW. The state of applied econometrics: Causality and policy evaluation. *J Econ Perspect*. 2017;31(2):3-32.

2. Robbins MW, Saunders J, Kilmer B. A framework for synthetic control methods with high-dimensional, micro-level data: evaluating a neighborhood-specific crime intervention. *J Am Stat Assoc*. 2017;112(517):109-126.

3. Lavrakas P. Encyclopedia of Survey Research Methods. 2008. doi:10.4135/9781412963947

4. Robbins MW, Davenport S. microsynth: Synthetic Control Methods for Disaggregated and Micro-Level Data in R. *J Stat Software; Vol 1, Issue 2* . January 2021. https://www.jstatsoft.org/v097/i02.

© 2021 Auty SG et al. *JAMA Health Forum*.

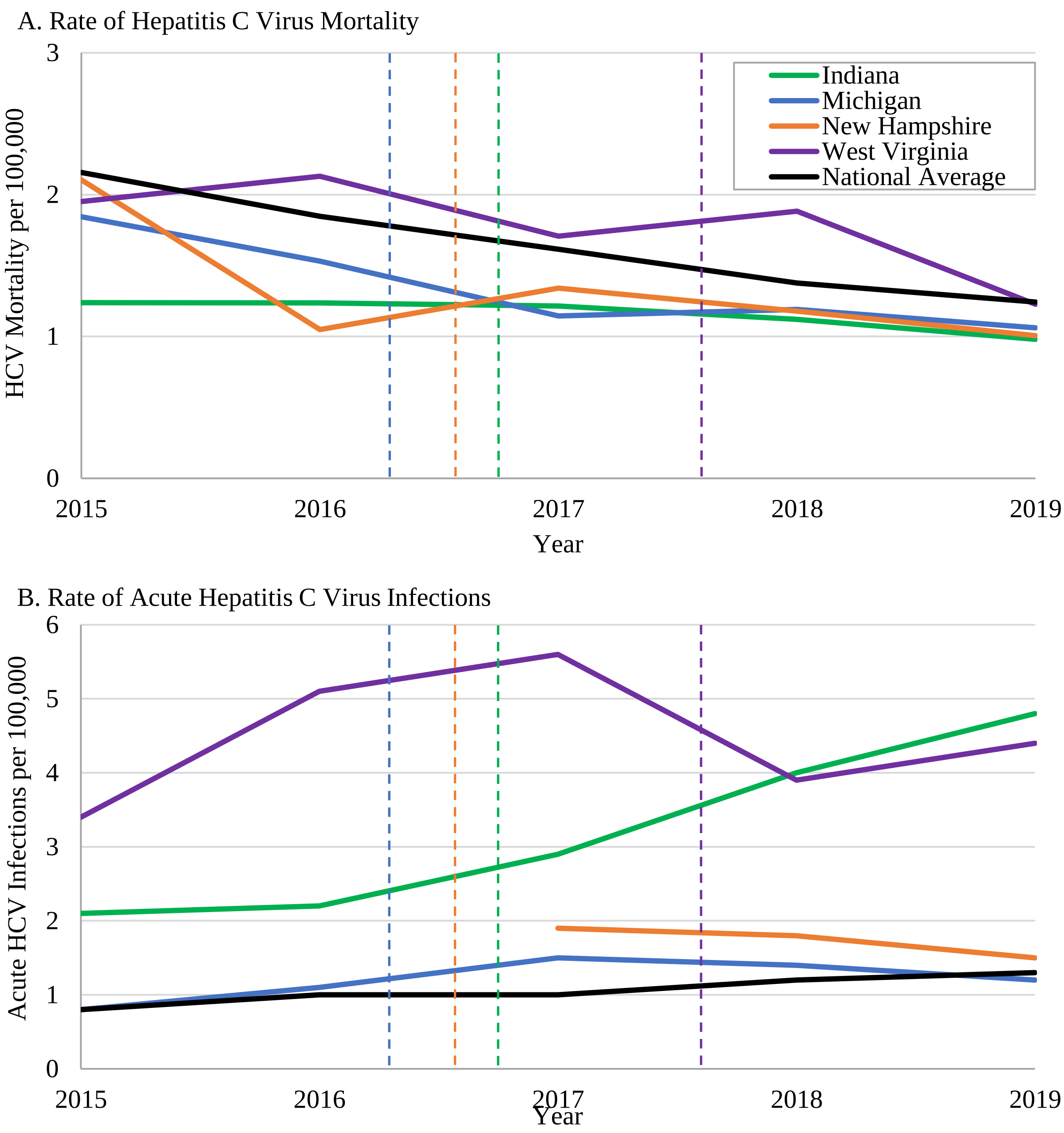

eFigure 1. Trends of Hepatitis C mortality per 100,000 residents in treated states and nationally.

Notes. Dotted lines indicate the quarter carve-outs occurred in treated states.

© 2021 Auty SG et al. *JAMA Health Forum*.

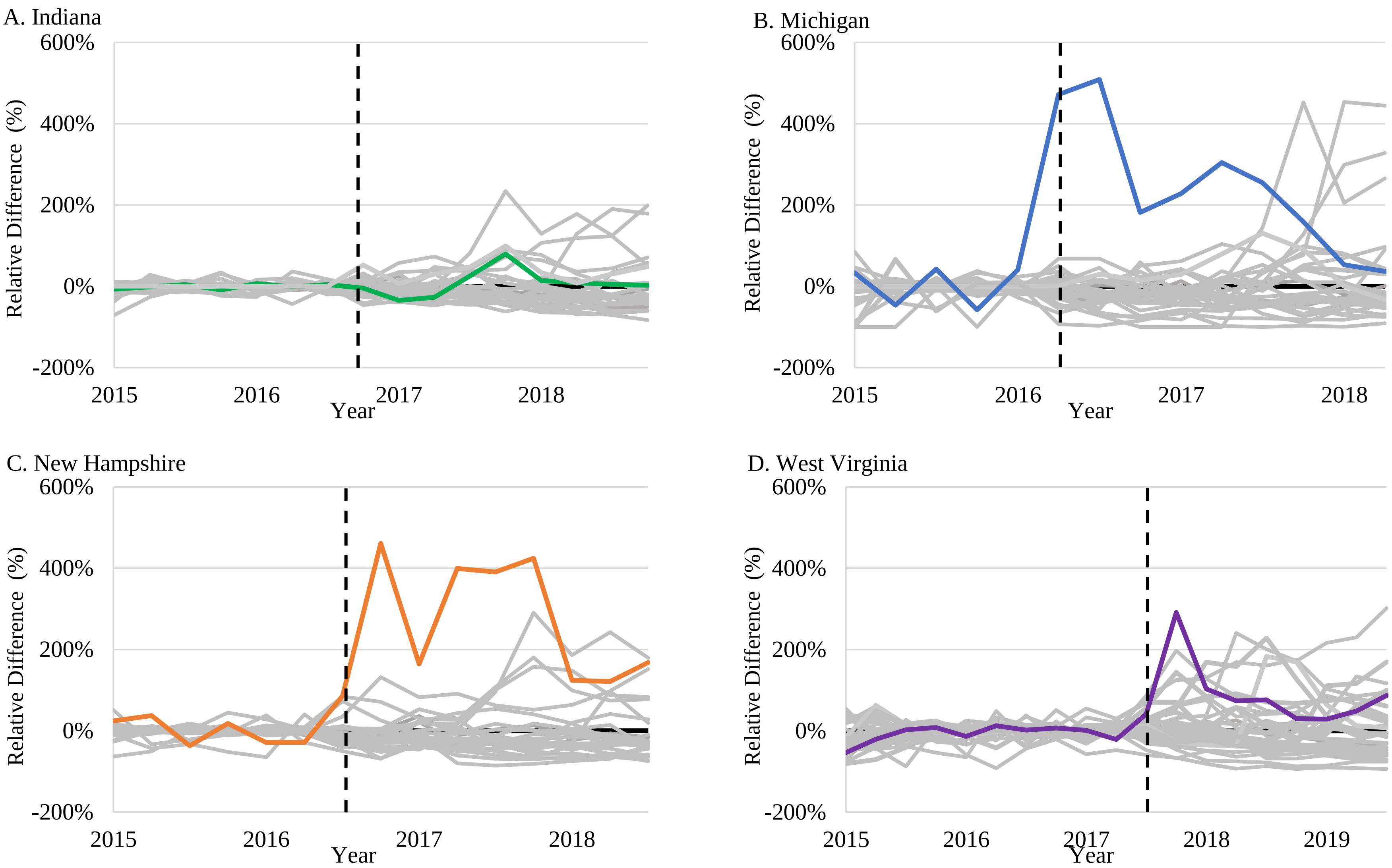

eFigure 2. Trends in HCV prescription fills per 100,000 enrollees per quarter for treated and individual control states.

Notes: Colored lines are treated states, gray lines are individual control states, and the dotted line indicates the beginning of the post period.

© 2021 Auty SG et al. *JAMA Health Forum*.
